# Shelf‐life enhancement of whole rainbow trout (*Oncorhynchus mykiss*) treated with Reshgak ice coverage

**DOI:** 10.1002/fsn3.636

**Published:** 2018-03-30

**Authors:** Samad Tavakoli, Mahmood Naseri, Elahe Abedi, Ahmad Imani

**Affiliations:** ^1^ Department of Natural Resources and Environmental School of Agriculture Shiraz University Shiraz Fars Iran; ^2^ Department of Food Science and Technology Fasa University Fasa Iran; ^3^ Department of Fisheries Faculty of Natural Resources Urmia University Urmia Iran

**Keywords:** phytogenic, rainbow trout (*Oncorhynchus mykiss*), Reshgak (*Ducrosia anethifolia*), shelf‐life

## Abstract

The effect of ice coverage comprised of Reshgak extract and Reshgak essential oil on shelf‐life extension of chilled whole rainbow trout (*Oncorhynchus mykiss*) was evaluated. Chemical (peroxide value (PV), thiobarbituric acids (TBA), total volatile nitrogen base (TVB‐N), and free fatty acids (FFA)), microbiological (total viable count (TVC) and psychrotrophic viable count (PVC)), and sensory evaluations (texture, color, flavor, and general acceptance) were investigated every 4 days during a 20‐day storage period. Results revealed that the effect of both icing systems led to considerably lower bacterial counts and chemical indices in comparison with the traditional ice coverage without such phytogenic. According to sensory analyses, fish stored in ice containing Reshgak essential oil had a longer shelf‐life (>16 days) and those stored in ice medium included with Reshgak extract possessed a shelf‐life of 16 days, whereas lot stored in traditional ice showed a shorter shelf‐life of 12 days.

## 1 INTRODUCTION

Rainbow trout (*Oncorhynchus mykiss*), as an important worldwide cold water fish species (Rezaei & Hosseini, 2008), is one of the main sources of protein, minerals, vitamins, and ω‐3 long‐chain polyunsaturated fatty acids (LC‐PUFAs) including eicosapentaenoic acid (EPA) and docosahexaenoic acid (DHA) (Abedi & Sahari, 2014; Woynarovich, Hoitsy, & Moth‐Poulsen, 2011). Seafood is reputable sources of LC‐PUFAs (Naseri, Abedi, Mohammadzadeh, & Afsharnaderi, 2013); however, they are very prone to oxidation and rancidity (Abedi, Naseri, Ghanbarian, & Vazirzadeh, 2016). The oxidation reactions can cause alterations in texture, color, and nutritional value of final product (Naseri & Rezaei, 2012; Naseri et al., 2013).

The efficiency of various methods of preserving seafood including low temperature, icing, and appropriate packaging, before addition of synthetic or natural antioxidants, has been used to control the development of undesirable changes in the product (microbial, enzymatic, and oxidative reactions) to extend its shelf‐life (Bensid, Ucar, Bendeddouche, & Özogul, 2014). Among the most common methods of seafood preservation, icing has gained considerable attention due to its cooling efficiency, temperature regulation, moisture retention, safety, ease of use, availability, and cost‐effectiveness (Shawyer & Medina Pizzali, 2003). Cold storage would effectively halt/hinder undesirable microbial, enzymatic, and oxidative reactions, whereas reduction in quality indices of fish does not stop under such conditions. Fish deterioration occurs depending on numerous factors such as storage temperature and period, fish size, and the nature of enzymatic degradation during chilling period (Abedi et al., 2016).

Plants extract and essential oil are well‐known bio‐preservatives (Bensid et al., 2014). They have been recently received considerable attention in seafood processing as alternatives for synthetic preservatives (Abedi et al., 2016; Álvarez, García García, Jordán, Martínez‐Conesa, & Hernández, 2012; Bensid et al., 2014; Li et al., 2012; Özyurt et al., 2012; Quitral et al., 2009). For instance, the inhibitory effects of essential oils and plant extracts on the microbiological and biochemical mechanisms involved in fish deterioration have been recently reported (Abedi et al., 2016; Álvarez et al., 2012; Li et al., 2012). It is conceivable that using plant bioactive compounds in cold and sanitary conditions such as ice coverage might be a promising method for increasing the seafood shelf‐life.

Recently, the efficacy of icing systems containing aqueous extracts of medicinal plants has been investigated in comparison with common ice coverage in few studies. The effect of ice contains thyme, oregano, and clove extracts on chemical, sensory, and microbiological indices of gutted and beheaded anchovy (*Engraulis encrasicholus*) was studied (Bensid et al., 2014). The results revealed that application of ice with plant extracts could improve the maintenance quality and shelf‐life of the fish. To the best of our knowledge, the efficacy of icing system containing plant essential oils to maintain fish quality during cold storage of whole fish has not been investigated.

Reshgak (*Ducrosia anethifolia* (DC.) Bioss.), a biennial herb of the Apiaceae family, is a wild‐growing medicinal plant growing wild in Iran, Afghanistan, Pakistan and Syria, Lebanon, Iraq, and some other Arab states and countries along the Persian Gulf (Hajhashemi, Rabbani, Ghanadi, & Davari, 2010; Mahboubi & Feizabadi, 2009; Mostafavi, Afzali, & Mirtadzadini, 2008). This aromatic herb has also been used to flavor food and beverage in traditional Iranian cuisine (Mostafavi et al., 2008). The main chemical components of Reshgak essential oil consisted of hydrocarbons, including α‐pinene, myrcene and limonene, and oxygen‐containing constituents, such as *n*‐decanal, *n*‐dodecanal, *n*‐decanol, trans‐2‐dodecenal, and cis‐chrysanthenyl acetate, with considerable antimicrobial properties against gram‐positive bacteria, yeasts, and some dermatophytes (Janssen, Scheffer, Svendsen, & Aynehchi, 1984).

There has been no scientific report concerning the effect of Reshgak extract (RE) and Reshgak essential oil (RO) on the shelf‐life of seafood. Modifying traditional preservation treatments such as ice coverage with reputable medicinal plants and spices including Reshgak seems a promising approach to extend the shelf‐life of chilled fish products. Therefore, the aim of this study was to investigate the possibility of extending seafood shelf‐life via RE and RO in icing system for transportation and chilled storage of rainbow trout. In this investigation, chemical, microbiological, and sensory changes of treated rainbow trout during chilled storage were assessed.

## 2 MATERIALS AND METHODS

### 2.1 Plant material (preparation extract and essential oil)

Dried Reshgak was acquired from a local market in Jahrom, Fars province, Iran, and botanically identified by Department of Horticulture Science, Shiraz University, Iran. The plant was grounded to a fine powder (Pars khazar, Iran). According to the method described by Makkar and Becker (1996), the plant powder macerated in ethanol 70% at a ratio of 1:5 for 48 hr. Finally, the filtered extract (Whatman filter paper no. 2) was concentrated using a rotary evaporator (Vacuum rotary, Fara azma, Iran) at 60°C.

The essential oil of the Reshgak areal parts was isolated via hydro‐distillation at Clevenger‐type apparatus for 4 hr. Using anhydrous sodium sulfate, Reshgak essential oil was dehydrated, and the essential oil was kept in refrigerator at 3 ± 1°C until subsequent usage (Mexis, Chouliara, & Kontominas, 2009).

### 2.2 Ice preparation

Preliminarily, various concentrations of RE (100, 200, 300, and 400 mg/L) and RO (500, 1,000, 1,500, and 2,000 mg/L) were examined. The effect of ice contains these concentrations on sensory and microbial changes of short‐term cold‐stored rainbow trout was studied. Concentrations with the best appearance lacking any strange odor, color, and having low microbial load were chosen (i.e., 300 mg/L RE and 1,500 mg/L RO). It is noteworthy that ice was prepared using distilled water. Also, traditional ice coverage was included in the experiment as a control group.

### 2.3 Raw fish, preparation, and processing

Rainbow trout (63 individuals; weight range: 350–370 g) were acquired from a local fish farm in Sepidan, Fars province, Iran. Using a traditional icing system, they were transported to the seafood laboratory, Department of Natural Resources and Environment, Shiraz University (2.5 hr). Immediately after arrival, samples were divided into three parts. Fish were placed in covered plastic box (20 kg capacity, white colored) and covered with a layer of corresponding ice treatments (RE, RO, or control) with a fish to ice proportion of 2:1 (w/w). Ice was renewed twice a day during storage period. All boxes were refrigerated in the same holding condition during a 20‐day experimental period. To conduct chemical, microbiological, and sensory assessments, samples were randomly taken every 4 days (i.e., days 0, 4, 8, 12, 16, and 20).

### 2.4 Chemical analyses

#### 2.4.1 Lipid extraction

Samples were subjected to chloroform/methanol/water (4:4:2.6 ratio) mixture to extract their lipid moiety according to the method described by Bligh and Dyer (1959). The wet tissue was homogenized with a mixture of chloroform and methanol in such proportions that a miscible system was formed with the water in the tissue. Dilution with chloroform and water separated the homogenate into two layers, the chloroform layer containing all the lipids and the methanolic layer containing all the nonlipids. A purified lipid extract was obtained merely by isolating the chloroform layer (Bligh & Dyer, 1959) using a rotary evaporator (Vacuum rotary, Fara azma, Iran) at 50–60°C.

#### 2.4.2 Peroxide value (PV)

The peroxide content of samples was determined according to Bensid et al. (2014). Fish oil was dissolved into chloroform–acetic acid mixture and subjected to an excess of iodide via a saturated solution of potassium iodide. The peroxides present oxidize the iodide to iodine and the iodine is then titrated to a colorimetric endpoint using sodium thiosulfate with starch as an indicator. Values were expressed as mEqO_2_ kg lipid^−1^.

#### 2.4.3 TBA value

Thiobarbituric acid reactive substances (TBARS) of lipid oxidation products were calorimetrically measured (Naseri, Rezaei, Moieni, Hosseini, & Eskandari, 2010). The method involves spectrophotometrically quantification of the pink‐colored complex resulted from the reaction of malondialdehyde (MDA) with 2‐thiobarbituric acid (TBA). TBA value was measured using 1‐butanol as the sole solvent. The absorbance is measured at 530 nm after heating to 95°C for 120 min and cooling. The TBA content of samples was expressed as mg MDA kg fish^−1^.

#### 2.4.4 Total volatile base nitrogen (TVB‐N)

Total volatile basic nitrogen (TVB‐N) of sample was determined according to Ojagh, Rezaei, Razavi, and Hosseini (2010). In brief, 2 g MgO was added to 10 g of homogenized fish samples in 400 ml distilled water. The distillate was collected using the 2% aqueous solution of boric acid and titrated using 0.05 mol/L sulfuric acid solution in the presence of the methyl red–methylene blue indicator as the endpoint detection sign. TVB‐N content of samples was expressed as mg N.100 g/fish.

#### 2.4.5 Free fatty acids (FFA)

Free fatty acids contents of samples were quantified via acidimetric titration of the Bligh and Dyer (1959) extract after addition of ethanol, in the presence of phenolphthalein as an endpoint indicator. FFA content was expressed as percentage of oleic acid (Naseri, Rezaei, Moieni, Hosseini, & Eskandari, 2011). Briefly, 1 g of fish oil was dissolved in 35 ml ethanol. The solution was titrated by NaOH (0.1 N) to the endpoint of the indicator (phenolphthalein), the pink color persistence for at least 30 s.

### 2.5 Microbial analyses

Samples (10 g each) were transferred to stomacher bags; 90 ml 0.1% peptone salt solution (NaCl, 0.85%, w/v) was added and homogenized for 1 min with a stomacher. Subsequently, serial dilutions were prepared using 0.1% peptone water and cultured on Plate Count Agar (PCA, Merck, Darmstadt, Germany). Total viable count (TVC) and psychrophilic viable count (PVC) were enumerated after incubation at 37°C for 2 days and 10°C for 7 days, respectively, and expressed as log_10_ CFU/g (Ojagh et al., 2010).

### 2.6 Sensory evaluation

At least six‐member trained panelists (three females and three males with age of 25 to 50 years old) performed sensory evaluations. Samples for organoleptic evaluation were prepared by steaming for 30 min at 60°C. The texture, taste, color, and overall acceptance were rated according to 5‐point hedonic scale (1, very poor to 5, excellent).

### 2.7 Statistical analysis

SAS software (version 9.0) was used for statistical analysis. Data, except for sensory analysis, were subjected to one‐way analysis of variance (ANOVA) followed by Duncan's multiple range test for getting the conservative differences between two means. The sensory data were subjected to nonparametric Kruskal–Wallis test, followed by Mann–Whitney *U* test for the identification of differences. All statistical analyses were interpreted at the significance level of *p* < .05. Data were presented at Mean ± *SD*.

## 3 RESULTS AND DISCUSSION

### 3.1 Chemical assessment

#### 3.1.1 Peroxide value

**Figure 1:**
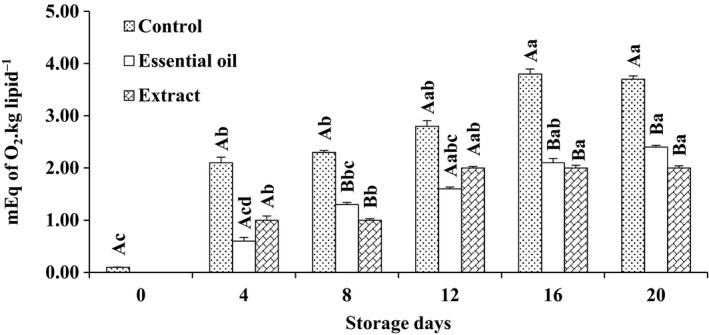
Changes in peroxide value of rainbow trout stored for 20 days in different icing media. Capital letters refer to comparison made among different treatments at each sampling time. Comparing the effect of storage time on each treatment was shown using lowercases. Different superscripts mean statistically significant differences (*p* < .05). A > B > C > D. (Extract, essential oil, and control depict samples stored in ice containing Reshgak extract, Reshgak essential oil, and those stored in icing medium without any additives, respectively)

The initial PV of fish samples lipid was 0.1 (mEq of O_2_ kg/lipid). The PV value of all samples significantly increased during storage (*p* < .05). As depicted in Figure 1, icing with RE and RO significantly hindered (*p* < .05) peroxide formation on days 8, 16, and 20. However, there were no significant differences (*p* > .05) among experimental groups on days 4 and 12 of the experimental storage of rainbow trout. The results revealed that the RE and RO were effectively slowed down lipid peroxidation of whole rainbow trout kept in bioactive icing system. According to the results, the inclusion of RE and RO in the icing system could reduce the formation of primary oxidation products. The previous research showed that major constituents of RO were α‐pinene, β‐myrcene, β‐pinene, and limonene. These components are increasingly considered as important antioxidant factors (Mottaghipisheh, Maghsoudlou, Valizadeh, & Arjomandi, 2014). Interestingly, RE showed the best antioxidant efficiency with the lowest PV values of samples. Our results were in good agreement with Quitral et al. (2009), and Bensid et al. (2014) studied the efficacy of icing with plant extract in reducing the formation of primary lipid oxidation products in Chilean jack mackerel (*Trachurus murphyi*) and anchovy (*Engraulis encrasicholus*), respectively.

#### 3.1.2 Thiobarbituric acid

**Figure 2:**
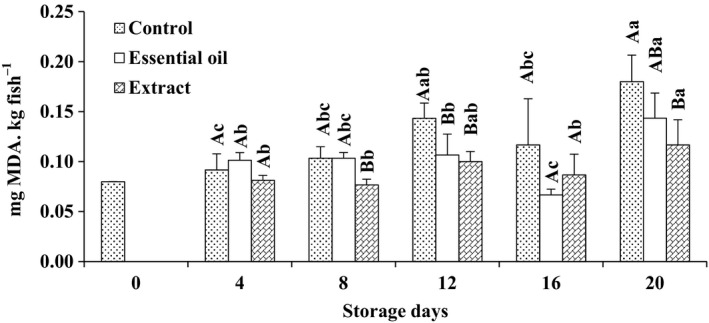
Changes in thiobarbituric acid value of rainbow trout stored for 20 days in different icing media. Capital letters refer to comparison made among different treatments at each sampling time. Comparing the effect of storage time on each treatment was shown using lowercases. Different superscripts mean statistically significant differences (*p* < .05). A > B > C > D. (Extract, essential oil, and control depict samples stored in ice containing Reshgak extract, Reshgak essential oil, and those stored in icing medium without any additives, respectively)

Thiobarbituric acid (TBA) is a common index of the extent of lipid oxidation at the second stage of auto‐oxidation, the stage of generation of aldehydes and ketones from peroxides (Fan, Chi, & Zhang, 2008; Jeon, Kamil, & Shahidi, 2002). Changes in TBA value of fish stored in different icing treatments were given in Figure 2. The initial TBA value of samples was low (0.08 mg MDA kg fish^−1^). However, it showed an increasing trend with storage time (*p* < .05). Meanwhile, TBA content of fish stored under icing system without any supplementary phytogenic was higher than samples stored in RE‐ or RO‐containing ice. The lipid oxidation products of all samples varied from 0.08 to 0.18 (mg MDA kg fish^−1^) with the highest values recorded in samples stored in common icing system at the end of the trial. It has been reported that plant extracts and essential oils may act as a high scavenger of radicals involved in lipid peroxidation protecting lipids from oxidation during storage (Kulisic, Radonic, & Milos, 2005). This antioxidant activity mainly has been related to flavonoids and polyphenolic compounds. All of these phenolic compounds have the ability to act as antioxidants by a free radical scavenging mechanism as well as ability to chelate transition metals such as iron to deactivate ionic forms (Martin, Frutos, Pérez‐Alvarez, Martinez‐Sánchez, & Tel Rio, 2002). However, TBA values of samples were well below the mean accepted values of TBA index for fish stored in ice (5–8 mg MDA. kg flesh^−1^) reported by Özyurt et al. (2012).

Comparison of TBA values of samples stored in RE‐ or RO‐supplemented ice with that of control group indicated a significant difference in secondary lipid oxidation product formation on days 8, 12, and 20 of experimental storage. The difference between RE and RO groups was only significant on day 8 of storage (*p* < .05). The lowest TBA index of RE group was indicative of its highest inhibitory potency against aldehydes and ketones formation in stored fish samples. The increase in TBA index of samples during the chilling storage might be due to the mild dehydration of fish and also to the augmented oxidation of unsaturated fatty acids (Fan et al., 2008). The increase in the TBA value of the present samples in the course of chilled storage was well in accordance to previous studies conducted by Abedi et al. (2016) and Gelman and Benjamin (1989) on rainbow trout and silver carp, respectively.

#### 3.1.3 Total volatile base nitrogen

**Figure 3:**
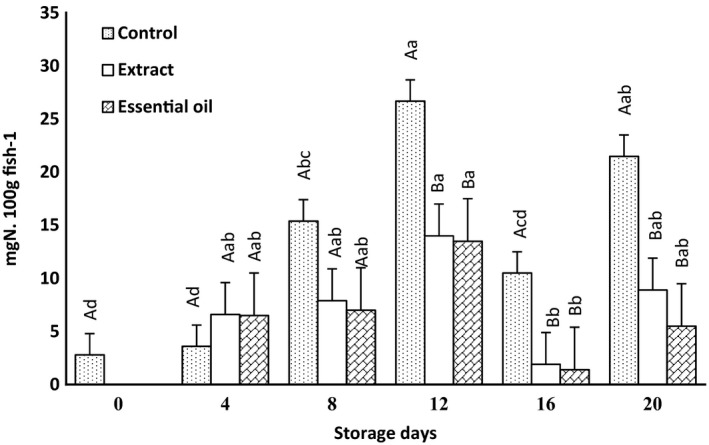
Changes in total volatile base nitrogen value of rainbow trout stored for 20 days in different icing media. Capital letters refer to comparison made among different treatments at each sampling time. Comparing the effect of storage time on each treatment was shown using lowercases. Different superscripts mean statistically significant differences (*p* < .05). A > B > C > D. (Extract, essential oil, and control depict samples stored in ice containing Reshgak extract, Reshgak essential oil, and those stored in icing medium without any additives, respectively)

TVB‐N index of various experimental groups during storage is given Figure 3. TVB‐N of fish at the start of the experiment was 2.8 mg N.100 g/fish. There was significant increase in TVB‐N contents of whole rainbow trout during ice storage (Figure 3, *p* < .05). TVB‐N value reached 21.5 mg N.100 g/flesh in control group, while those of RE and RO groups was 8.9 and 5.1 mg N.100 g/flesh by the end of storage. TVB‐N values increased with different rates depending on the nature of treatments. This may be attributed to the breakdown of proteins as a result of activity of microbial strains and proteolytic enzymes. The highest TVB‐N contents of all experimental groups were observed on day 12 of storage, corresponding to sensory rejection of those fish stored in traditional icing system. Icing system containing RE and RO had significantly lower TVB‐N values of 13.5 and 14 mg N.100 g/flesh, respectively, in comparison with fish kept under common icing system (26.7 mg N.100 g/flesh on day 12 of storage). The TVB‐N content of control samples on day 12 of storage was over the limit of acceptability of 25 mg N.100 g/flesh (Gimenez, Roncales, & Beltran, 2002; Ojagh et al., 2010).

The statistical analysis revealed a considerable difference on days 12, 16, and 20 of storage with regard to TVB‐N (*p* < .05) among various experimental groups, while no significant differences were observed on days 0, 4, and 8 days in this respect. There were no significant differences between RE‐ and RO‐supplemented ice regarding the criterion through storage time (*p* < .05). This may be attributed to the role of such compound on microbial population and bacterial growth as antimicrobial agents.

#### 3.1.4 Free fatty acids

**Figure 4:**
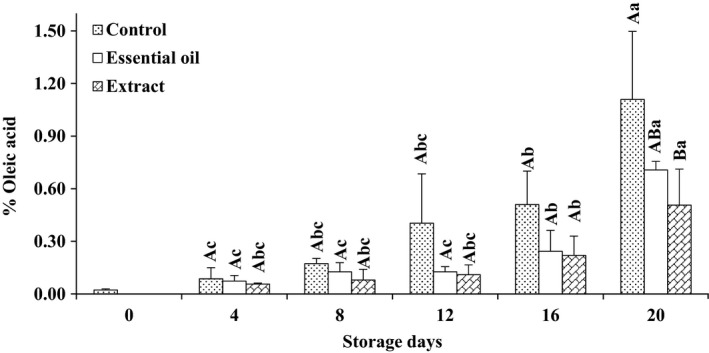
Changes in free fatty acid value of rainbow trout stored for 20 days in different icing media. Capital letters refer to comparison made among different treatments at each sampling time. Comparing the effect of storage time on each treatment was shown using lowercases. Different superscripts mean statistically significant differences (*p* < .05). A > B > C > D. (Extract, essential oil, and control depict samples stored in ice containing Reshgak extract, Reshgak essential oil, and those stored in icing medium without any additives, respectively)

Free fatty acids (FFA) are products of enzymatic disintegration of lipids (Uçak, Özogul, & Durmuş, 2011). The FFA contents of chilled stored rainbow trout were depicted in Figure 4. The lowest FFA index belonged to samples stored in ice supplemented by RE, while the highest FFA value was measured in control group (*p* < .05). FFA content of fish kept in traditional ice significantly differed from that of group stored in ice prepared with RE on day 20 of storage (*p* < .05); however, fish stored in ice containing RO was intermediate in this regard (*p* > .05). Lower FFA contents of fish stored in ice supplemented with phytogenic might be due to the reduced rate of lipid hydrolysis in comparison with common icing system. Ashton and Bremner (2002) hypothesized that FFAs are more prone to oxidation in comparison with esterified fatty acids. Our results were in agreement with reports by Ibrahim and El‐Sherif (2008) and Emir Çoban (2012) on frozen tilapia and rainbow trout, respectively.

### 3.2 Microbial assessment

**Figure 5:**
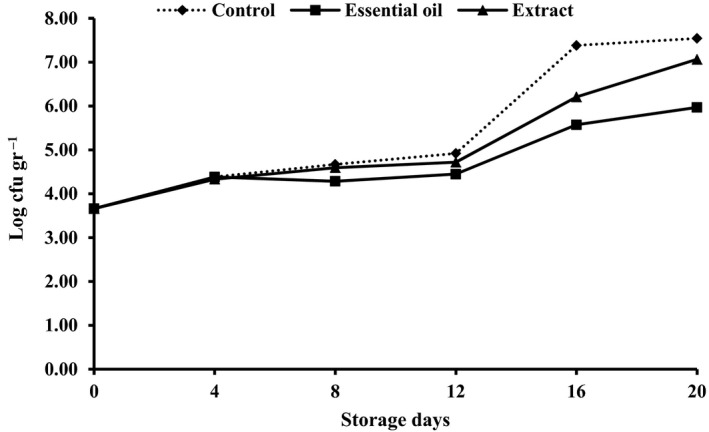
Changes in total viable counts (TVCs) of fish samples during chilling storage

Total viable counts (TVCs) of rainbow trout during chilling storage under different icing conditions are shown in Figure 5. The initial total viable bacterial counts indicated an acceptable fish quality (3.66). TVC of rainbow trout stored in traditional ice exceeded 7 on day 16 of storage. Fan et al. (2008) have already reported an increase in TVC of fish during ice storage. On the other hand, TVC of samples stored in ice included with RE exceeded 7 on day 20 (i.e., 4 days later). However, TVC of samples stored in ice containing RO was 5.97 at the end of the storage period, still well below 7, the maximal recommended limit of 7 for TVC of raw fish (Ojagh et al., 2010).

**Figure 6:**
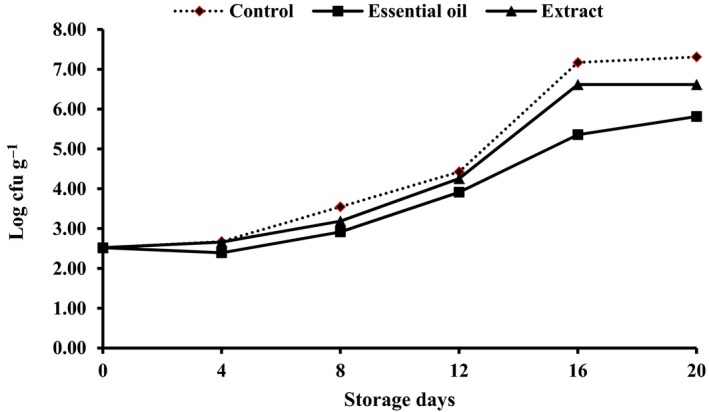
Changes in psychrophilic viable counts (PVCs) of fish samples during chilling storage

Psychrophilic bacteria are the major group of microorganisms responsible for aerobic spoilage of chilled stored fish (Ojagh et al., 2010). In the present study, the initial PVC was 2.52 (Figure 6). Furthermore, the variation of PVC of samples was similar to that of TVC during the chilled storage; control group showed the highest value on day 20 (7.31), followed by samples stored in ice prepared with Reshgak extract (6.62), and the lowest count (5.81) has been observed in samples stored in ice prepared with RO.

Results of microbial assessment indicated the microbiological shelf‐life up to 12 days for the control samples, 16 days for samples stored in ice prepared with RE, and more than 20 days for samples stored in ice containing RO.

The subsequent sensory shelf‐life determination indicated that in common icing system, the fish were acceptable up to 12 days, while in RE icing system, fish were consumable up to 16 days. However, including RO in ice resulted in even extended shelf‐life up to 20 days (Table 1). These findings implied that achieving longer shelf‐life for whole rainbow trout is possible by including phytogenic ingredient such as RE and RO with antioxidant and antibacterial properties into icing system. Several authors have similarly shown that herbal extracts and essential oils have significant inhibitory activities toward pathogenic and spoilage microorganisms (Hernández, García García, Jordán, & Hernández, 2014; Li et al., 2012; Sant'Ana & Mancini‐Filho, 2000).

**Table 1:** Sensory indices changes during chilling storage

Sensory index	Treatment	Sampling days				
		0	4	8	12	16
Texture	Control	Aa	Ba	Bb	Bc(SD)	Bd(SD)
	Essential oil	Aa	Aa	Aa	Ab	Ac
	Extract	Aa	Aa	Ab	Ab	Ac
Color	Control	Aa	Aa	Bb	Bb	Cc(SD)
	Essential oil	Aa	Aa	Aa	Aa	Ab
	Extract	Aa	Aa	Aa	Cb	Bb
Flavor	Control	Aa	Bb	Bc	Bd(SD)	Bd(SD)
	Essential oil	Aa	Aa	Aa	Ab	Ab
	Extract	Aa	Aa	Aa	Bb	Bb(SD)
General acceptance	Control	Aa	Aa	Ba	Cb(SD)	B(SD)
	Essential oil	Aa	Aa	Aa	Ab	Ac
	Control	Aa	Aa	Cb	Bc	Ad(SD)

Comparison of different treatments is shown in capital letters.

The effect of storage time on each treatment is shown in lowercase letters. (*p *<* *.05). A > B > C > D. SD means rejected as spoiled sample.

Extract means, samples stored in ice containing Reshgak extract; essential oil means, samples stored in ice containing Reshgak essential oil.

One might infer that antimicrobial potency of RE was less than RO in preserving rainbow trout during chilled storage. According to Figures 5 and 6, higher bacterial counts were enumerated in samples stored under traditional ice coverage followed by samples stored in ice containing RE and RO. Possibly these results are related to the bioactive compound such as polyphenolic components of the essential oil, namely flavonoids (Negi, 2012). According to previous results published by Mahboudi and Feizabadi (2009), decanal or decyl aldehyde, α‐pinene, undecanal, and thymol were the main constituents of the RO. These elements showed antimicrobial activity against clinical isolates of *Staphylococcus aureus*, including methicillin‐resistant (MRSA) and methicillin susceptible of *S. aureus* (MSSA). Other research proved that the main oxygen‐containing aliphatic components of the RO have a remarkable antimicrobial activity against gram‐positive bacteria, yeast, and some dermatophytes (Janssen et al., 1984).

### 3.3 Sensory evaluation

Sensory assessment has been an efficient method of evaluating the quality change of a product over storage time (Abedi et al., 2016). Sensory characteristics including texture, color, flavor, and general acceptability of steam‐cooked samples are shown in Table 1.

At the beginning of storage period, the texture was firm and flexible; however, fish kept in ice without any phytogenic supplementation showed a soft and undesirable texture and determined as spoiled sample (SD) by the experiment panelists on day 12 of storage (Table 1). Texture of fish stored in ice containing RE or RO was acceptable, although there were no significant differences between their textures until the end of experiment (day 16). Higher texture quality of Reshgak‐treated samples might be due to reduction in chemical and microbial deterioration of such samples. Color changes of all samples were obvious and significant (Table 1). Color change was significantly different among treatments on days 8, 12, and 16 of storage. The most acceptable color belonged to samples stored in ice containing RO followed by samples stored in ice containing RE and control groups, respectively. Color of control groups was not acceptable on day 16 of testing and regarded as spoiled sample (SD) by panelists. The changes in flavor were significant especially in samples stored in ice containing REs and control group. On days 4 and 8, there were significant differences between control group and fish stored in ice containing REs. Considering the flavor of control group and samples stored in ice containing RO, there were significant differences between days 4, 8, 12, and 16 of sensory assessments; however, RE and RO groups differed only on days 12 and 16 of storage. Therefore, it is inferable that ice contains RO had a positive impact on the panelists, while ice contains RE exhibited a negative impact on panelists on days 12 and 16 of assessments. It could be due to domination of the flavor of RE compounds to the fish meat flavor. The flavor of control group and those fish stored in ice containing RE were rejected by panelists on days 12 and 16, respectively. The adverse changes in terms of general acceptability of control and those fish stored in ice containing RE were significantly more pronounced than fish kept in ice containing RO. According to Table 1, samples stored in ice supplemented by RO showed the highest general acceptance on days 8, 12, and 16 of assessments. The general acceptance of control group and samples stored in ice containing RE were rejected by panelists on days 12 and 16, respectively. According to the results of chemical and microbial analysis (Figures 1, 2, 3, 4, 5, 6) as well as sensory test (Table 1) during storage, quality attributes of the products were deteriorated due to lipid oxidation and bacterial growth which have been the main factors that determining fish quality loss and shelf‐life reduction. Lipid oxidation leads deterioration of lipids and proteins decay which, in turn, contribute to the reduction in nutritional quality as well as deterioration in flavor, color, and texture of final products.

## 4 CONCLUSION

The proposed bio‐preservatives–icing system with Reshgak extract and essential oil proved to be effective for fish preservation mainly due to their antioxidant and antimicrobial properties. Sensory evaluation also revealed that such icing system might significantly extend the seafood shelf‐life. The ice supplemented with Reshgak essential oil would extend the shelf‐life of whole rainbow trout for nearly further 8 days, and inclusion of extract of the plant would extend the shelf‐life of rainbow trout for another 4 days in comparison with traditional icing medium without any phytogenic material. Reshgak extract and essential oil combined with ice seem to be a promising solution for extending fish shelf. These results may also be of practical interest for on‐board goals.
